# In-home versus hospital preoperative balance and proprioceptive training in patients undergoing TKR; rationale, design, and method of a randomized controlled trial

**DOI:** 10.1186/s12891-017-1887-4

**Published:** 2017-12-08

**Authors:** José-María Blasco, Celedonia Igual-Camacho, Sergio Roig-Casasús

**Affiliations:** 1 Department of Physiotherapy, University of Valencia, Calle Gascó Oliag 5, 46010 Valencia, Spain; 2 Group of Physiotherapy in the Ageing Process, València, Spain; 3 Joint Research Unit La Fe-UV (IRIMED), València, Spain; 4 Hospital Clínico y Universitario de Valencia, Avenida de Blasco Ibáñez, 17, 46010 València, Spain; 5 Hospital Universitario y Politécnico de La Fe, Avenida de Fernando Abril Martorell, 106, 46026 València, Spain

**Keywords:** Total knee replacement, Total knee arthroplasty, Balance, Proprioception, Sensorimotor, Preoperative intervention, Physiotherapy

## Abstract

**Background:**

Severe knee osteoarthritis, as well as the surgical procedure of total knee replacement that aims to reduce its symptoms, cause great deterioration on the proprioceptive system. Taking this fact into account, and considering that balance abilities positively influence the capacity to perform basic functional tasks, this trial aims to find the short and mid-term effects of a preoperative balance and proprioceptive training when conducted by patients undergoing total knee replacement. Along with the effects, it is intended to determine whether in-home based training can be as effective as hospital training. The results will help to conclude whether the possible benefits may outweigh the health costs.

**Methods:**

Seventy-five participants will take part. The trial will include in-home and supervised hospital experimental training compared to a non-active control group in order to estimate the actual effect of the proposal against the benefits due exclusively to the surgical procedure. Interventions last 4 weeks prior to surgery, and the follow-up will be at 2w, 6w, and 1y following the operation. The primary outcomes are in agreement with the goals: self-reported functionality in terms of KOOS and overall balance in terms of the Berg Balance Scale. The secondary outcomes will include the measurements of static and dynamic balance abilities, pain, function, and quality of life.

**Discussion:**

It is expected for the results of this trial to provide relevant information in order to decide if a specific intervention is cost-effective to be implemented in clinical practice.

**Trial registration:**

Clinicaltrials.gov identifier NCT03100890. Registered in April 4, 2017.

## Background

Total knee arthroplasty is a solution for severe osteoarthritis in advanced stages, when functional disability and especially pain are difficult to alleviate with conservative treatment and drugs. The aging of the population, the high prevalence of the condition, the progress in surgical techniques, the level of satisfaction, along with the increase of the quality of life of the patient, are factors that have made this a common procedure [1, 2].

In spite of the overall satisfactory outcomes, some patients may present pathological gait patterns, difficulty in performing basic functional tasks, and in maintaining balance and postural control, even one year after surgery [3–5]. In order to optimize outcomes, the procedure ought to be accompanied by physiotherapy. Systematically, an early postoperative intervention is recommended [6]. Also, while it is true that the literature is not conclusive regarding mid and long-term effects [7], a preoperative program is always advisable, can optimize early results, and reduce the length of stay [8]. By contrast, it is time consuming and expensive, thus being necessary to conduct cost-effectiveness analyses that justify the use of such program [8–10].

Physiotherapy generally focuses on strengthening exercises and functional performance based trainings. Balance and proprioception work is usually included in training programs, but to a lesser extent. However, the recovery and enhancement of these aspects may be a determining factor, since good balance abilities positively influence the capacity to perform basic functional tasks [11]. Taking this relationship into account, and that both the condition itself and the high invasiveness of the surgical procedure involve a significant deterioration of the proprioceptive system [12], seems interesting to appraise a proposal of intervention which begins in the preoperative phase, and looks for benefits not only among the muscular and functional outcomes, but also in sensorimotor terms.

Balance and proprioceptive approaches have been referred to as sensorimotor and neuromuscular trainings. A recent qualitative review suggested that these may be an acceptable adjunct to usual care in physiotherapy [13]. Nevertheless, the literature is scarce with respect to the impact of an additional training specifically oriented towards the recovery of balance and proprioception. Gstoettner et al. [5] suggested that balance and proprioceptive training is effective in improving standing balance, can avoid pathological gait patterns and falls, and proposed that further investigations were needed in order to give consistency to the findings. Later, Hubber et al. [14] and Villadsen et al. [15] evaluated the effects of a preoperative neuromuscular training program, while Fernandes et al. [16] analyzed the cost-effectiveness of the latter. None of them performed a comparative study between hospital and in-home intervention appraising the effects, pragmatism and costs.

With this background, this trial will determine the short and mid-term effects of a preoperative balance and proprioceptive training when conducted by patients undergoing total knee replacement, and the impact that this may have on the balance of the patient, functionality and quality of life [17]. Along with the effects, it is intended to determine whether in-home based training can be as effective as hospital training. The results will help to conclude whether the possible benefits may outweigh the health costs.

## Method

### Design and ethics

This is a 3-arm randomized trial, which is carried out prospectively at Hospital Universitario y Politécnico La Fe of Valencia, Spain. The trial has been designed in accordance with the guidelines proposed in the Helsinki Declaration, was approved by the Ethical Committee for Clinical Research and by the Scientific Committee of the mentioned hospital, and was prospectively registered in April 4, 2017 (clinicaltrials.gov identifier NCT-03100890).

Potential participants must give express consent for the use of data for exclusively scientific and research purposes. The data are anonymized from the origin, with initials and numerical identifiers for the electronic bases. The data will be extracted by the evaluators and managed exclusively by the principal investigator of this project, who will be the only one with access to the complete database. The database will be kept in the Physiotherapy Department of the University of Valencia, subject to the center’s security protocols.

### Participants

**Fig. 1:**
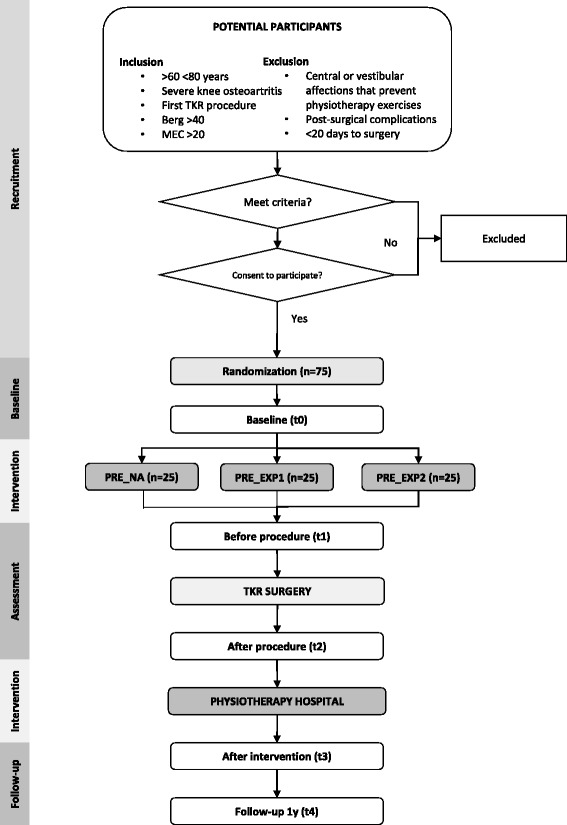
Participants flow chart

Potential participants are invited to participate by the orthopedic surgeon, who performs an initial check of whether the criteria are met. The criteria will include those subjects over 60 years of age, with severe knee osteoarthritis, who will undergo their first surgery for total arthroplasty, and present a low risk of falling with a score of over 40 on the Berg Balance Scale [18] and a moderate cognitive condition to adequately follow the interventions, with more than 20 points in the Spanish version of Lobo of the Mini-Mental State Examination [19]. In case the surgeon refers the potential participant to the rehabilitation department for possible inclusion, the last 2 criteria are evaluated by the rehabilitators. Criteria exclude those subjects with central or vestibular affections that directly affect their balance, or with postoperative complications as possible infections. If the potential participant meets the criteria, reads and understands the information sheet, and signs a consent form, become a participant in the trial. Those potential participants on a waiting list whose date of surgery is established for less than 20 days are not considered for inclusion. In the case of modifications of the date of surgery, the participant will continue forming part of the study, as long as the date is not modified beyond 30 days. The participants flow chart is shown in Fig. 1.

### Sample size

A priori sample size estimation was made with the tool G*Power3.1 [20]. One-way ANOVA power analysis with three groups was based on the results of previous works [21]. The analysis considered that the groups receiving preoperative intervention will reach a score on the primary outcome the Berg Balance Scale of 53, this score being 5 points above that of the non-intervention group, with a reasonable standard deviation of 4.5 points, and with α = 0.05. The trial must consist of groups of 20 participants in order to achieve *d* > 0.8 and *f* ^2^ > 0.5. Predicting over 20% of sample loss, a total of 75 participants will be divided into 3 equal groups. Once data are collected, a post-hoc analysis will determine if the sample provided sufficient power to the trial.

### Blinding and randomization

Participation occurs sequentially. At the time that a subject has an established date of operation, the physician checks the criteria are being met. If so, the potential participant is informed of the study and is referred to the rehabilitation department where, if is definitely admitted will take part in the randomization process. The randomization is generated from the output of the Matlab® statistical program by external assessor. A physiotherapist with 3 years of experience and blinded to the intervention groups will be in charge of the outcome assessment. The blinding of the participants is not assured, as they are informed about the trial before randomization. Neither is it the blinding of those who provide the treatments for the same reason. However, physiotherapists do not take part in the process of randomization and participant allocation. The external assessor in charge of the randomization is blinded to the intervention groups that have a numerical coding, and will receive the data of the participants for the statistical processing. The interpretation of the results will be carried out by the whole team.

### Interventions

The interventions are supervised by 2 physiotherapists with more than 15 and 20 years of experience. All groups perform the same postoperative intervention. This consists of a standard recovery program based on the Brotzman principles [22] and clinical experience, and have been pragmatically adapted to hospital standards according to the following aspects: muscular strengthening with isometric, isotonic, and resistance exercises, functional exercises, and to a lesser extent proprioception and balance exercises. The protocol consists of 3 phases: a warm-up phase with passive, active-assisted, and active mobilizations, a work phase with functional exercises for balance and strength, and a cooldown phase with relaxation activities. The sessions last approximately 60 min, and the loads are adapted to the possibilities of the subjects to avoid fatigue. A total of 12 sessions are prescribed, on alternate days, 3 times a week, and in a hospital setting. The intervention begins in week 2 and ends in week 6. In the case that the physician considers that a participant should perform a greater number of sessions, the standard hospital protocol continues. However, all participants are always evaluated after session 12 and during week 6, as the interventions may have influenced the early postoperative outcomes.

Preoperative and control interventions are detailed below, while the distribution of the interventions in groups are shown in Table 1.

**Table 1:** Interventions and groups

Intervention/group	Non-active comparator	Experimental 1	Experimental 2
Preoperative			
In-hospital preoperative		x	
In-home preoperative			x
Postoperative			
Conventional physiotherapy	**x**	**x**	**x**

#### In-hospital supervised preoperative intervention (PRE_ EXP1)

Different exercises are proposed and developed over the course of the 12 sessions on alternate days, divided in 4 weeks of training. On the one hand the intervention includes a standard program of rehabilitation with strengthening exercises. In addition, each session includes a total of 4 to 5 balance and proprioceptive exercises performed over a period of 20 min. The training has been based on the Fitzgerald principles to train proprioception, on previous of our works, and on clinical experience [21, 23]. The first 2 weeks are oriented to enhance balance and proprioception with the side, treadmill, cross, and tandem walk exercises. These exercises are excluded in weeks 2 and 3 and multiple changes of direction, foam activities and balance work with the Bohler plate are performed instead. Also 5 min training of weight bearing and testing the limits of stability are performed during the last 4 sessions. Progression in the exercises is implemented as tolerated, always trying to avoid fatigue. The physiotherapist is responsible for recording attendance at rehabilitation sessions.

#### In-home preoperative intervention (PRE_EXP2)

The in-home exercise program is essentially based on the same principles than PRE_EXP1, but adapted so it can be conducted at home. The first day is an education with the explanation of the exercises. The training is carried out in the hospital and is supervised by the physiotherapist. The participant is asked to repeat the exercises, and possible doubts are resolved. The intervention consists of a phase with exercises to strengthen the main musculature of the lower limb, and a second phase of balance and proprioceptive work. The muscular enhancement exercises are isometric quadriceps in extension, elevations of the lower limb with knee in extension, ankle in dorsal flexion, and slight hip flexion combined with abduction and adduction. Additionally, the middle gluteal and open chain quadriceps are also performed. Each set of exercises is performed in 4 series consisting of 10 repetitions with a rest of 90 s between each series. The proprioception exercises consisted of tandem position and walking, single-leg position, and treadmill walking. Each exercise has to be performed for 2–4 min. And the participants were asked to do so safely, for instance in a hallway or in front of a table or chair, to avoid falls. After session 6, the participant is appointed for a reminder and verification of exercises, as well as possible corrections in the implementation of the intervention. The participant is requested to register the number of sessions performed in a calendar. The physiotherapist phones the participants on a weekly basis, in order to check both the compliance of the program and the calendar fulfillment.

#### Non-active preoperative intervention (PRE_NA)

In order to evaluate the true effect produced by the interventions, and to determine whether the postoperative results obtained are due to the appropriateness of the proposals, a non-active control group (third arm) was added.

### Reproducibility of the interventions

The procedures were standardized and pilot tested to ensure participant recruitment, outcome evaluation, data handling and management, and safety. A verification of the procedures is planned for periods of 6 months, through observation and meetings to ensure the reproducibility of the protocols and their consistency.

### Outcomes

#### Summary of outcomes and prioritization

In order to characterize de sample and test potential predictors, the sample descriptors and the baseline characterization in terms of the demographic and biomedical characteristics are extracted, including comorbidities.

The following outcome measures are of interest in this trial, according to the proposed hierarchy.

- **Primary outcomes**

- Self-reported function in daily living as measured with the Knee Injury Osteoarthritis Outcome Score (KOOS) [24].
- The overall state of balance in terms of the Berg Balance Scale [18].

b. **Secondary outcomes**

- Knee function in terms of the range of motion in flexion and extension and the isometric quadriceps strength.
- Abilities in terms of dynamic and static balance, as well as testing the limits of stability, as measured with the Timed Up & Go Test, the Single Leg Standing Balance Test, and the Functional Reach Test respectively.
- Pain, assessed with the Pain subscale of the KOOS scoring system.
- Quality of life and preoperative anxiety, assessed with the health-related questionnaire the EuroQoL-5D and the corresponding subscale of the KOOS scoring system.

c. **Other outcomes**

- Economic evaluation. Cost-effectiveness analysis.
- Qualitative evaluation: Patient expectations and caregivers opinion.

#### Outcomes description

**KOOS** is a self-reported questionnaire developed as an instrument to assess the patient’s opinion about their knee and associated problems. It evaluates the short-term and long-term symptoms and function in subjects with knee injury and osteoarthritis. The KOOS holds five separately scored subscales: Pain, other Symptoms, Function in daily living (ADL), Function in Sport and Recreation (Sport/Rec), and knee-related Quality of Life (QOL). A normalized score is calculated for each subscale, being 100 no symptoms and 0 extreme symptoms [24]. All subscales are evaluated in this research, but Sport/Rec. The ADL scale is set as primary outcome, together with the Berg Balance Scale. All other scales are considered secondary outcomes.

The **Berg Balance Scale**, is used to assess balance abilities in terms of the overall state of balance. It is a 14-item questionnaire administered in order to measure balance among older people with impairment in the balance function by assessing the performance of functional tasks [18].

The **Timed Up & Go Test,** used to assess dynamic balance, is a test of general mobility, in which the individual rises from a standard arm chair without using their arms, walks 3 m, turns around, returns to the chair, and sits down [25]. The test score measured in seconds is taken as the mean of 2 trials.

The **Single Leg Standing Balance Test** is a simple method to assess static balance. It measures the time that a patient can hold their balance on just one leg. This outcome could not be assessed after the procedure, due to the medical prescription, since it is too early to achieve one-legged support.

The **Functional Reach Test**, used to assess stability, is a test to detect balance deterioration and changes in performance over time, measures the maximum distance the participant could reach forward while standing in a fixed position. The procedures are adapted based on those described by Duncan [26].

##### Knee function range of movement (ROM)

The joint function in terms of flexion and extension range of movement will be measured with a manual goniometer. The participants are placed in a supine position while the hip was bended at 90 degrees.

##### Knee function strength

For the measurement of the strength of the quadriceps, a dynamometer Manual Muscle Tester 01165 Lafayette Instrument® is used. The isometric force of both quadriceps is measured for 6 s. The test is performed 3 times and the mean is calculated. The maximum peak force data are also extracted.

##### EuroQoL-5D

This is a questionnaire administered to measure the health-related quality of life. The descriptive system contains 5 dimensions of health [27]. It is important to note that preoperative anxiety is being assessed with item number 5 of this test.

##### Pain

This will be assessed with the Pain subscale of the KOOS scoring system [28].

#### Other outcomes

##### Qualitative evaluation of patient expectations

The evaluation will be based on the anchor methods, asking the participant about their expectations of the benefits that they hope to achieve due to the surgical intervention and following their participation in the trial. The questions will be answered with a Likert scale. Subsequently, the fulfillment of these expectations will be verified.

##### Economic evaluation

Cost-effectiveness analysis, which is further explained in the data analysis section.

#### Outcome assessment

The baseline assessments will be carried out by a physiotherapist blinded to the interventions, following the signing of an informed consent form and prior to the start of the physiotherapy sessions. The proposed time for the outcome evaluation is in the preoperative period (baseline, between 8 and 4 weeks), before (1 week) and after (2 weeks) the surgical procedure, after the postoperative intervention (6 weeks) and 12 months follow-ups. Details are given in Table 2.

**Table 2:** Detail of variables and times for evaluation of outcomes

Time point	Enrolment	BL	BP	AP	AI	12 m
Eligibility screen	x					
Informed consent	x					
Randomization	x					
Assessments						
Demographic and biomedical	x					
Primary						
Self-reported functionality	KOOS	x	x	x	x	x
Balance (overall)	Berg Balance Scale	x	x	x	x	x
Secondary						
Function	ROM (flexion)	x	x	x	x	x
	ROM (extension)	x	x	x	x	x
	Strength	x	x	x	x	x
Balance (dynamic)	Timed Up & Go	x	x	x	x	x
(static)	One-leg Stand	x	x		x	x
(stability)	Functional Reach	x	x	x	x	x
Quality of Life	EuroQoL	x			x	x
Anxiety	EuroQoL(5D)	x	x		x	
Cost-effectiveness						
	Qaly					x
Patient expectations						
		x			x	x
Interventions		BL	BP	AP	AI	12 m
Non-active comparator				x------x		
In-hospital preoperative		x------x		x------x		
In-home preoperative		x------x		x------x		

*BL* Baseline, *BP* Before procedure, *AP* After procedure, *AI* After intervention, *KOOS* Knee Injury Osteoartritis Outcome Score, *ROM* Range of Movement in degrees, *Qaly* Quality adjusted life year

### Data analysis

The implementation of protocols will be presented with descriptive statistics. In an exploratory way, the drop-out ratios will be estimated and the survival analyses will be used [29]. This aspect will also be taken into account throughout the follow-up and registering when drop-outs occur. The incidence of adverse effects at 6 weeks will be evaluated, which will be categorized according to whether they are considered to be definitive, probable or possibly related to the interventions.

It is hypothesized that the experimental groups will have enhanced balance abilities, which may also have a direct impact on functionality in the short and mid-term. All temporal instants are of interest, although particular attention will be paid to the early postoperative effects (6w) and maintenance of potential mid-term benefits (1y).

The data analysis will include the sample descriptors, a comparison of the baseline condition with the t tests that will be parametric or not depending on the results of the tests of Wilcoxon and Shapiro-Wilk, and a comparison of the progress due to the results of the intervention with an ANOVA analysis of repeated measures, looking for interactions initially as a function of time, group, and time per group. In case of baseline differences between the participants, an ANCOVA analysis will be conducted instead, in which the data will be adjusted to the baseline scores. Analyses will be performed for primary and secondary outcomes. Post-hoc analyses will be performed accordingly. The significance levels will be set at 95%.

A qualitative analysis will be carried out in which it will be estimated if the obtained progressions measured as the mean values of the difference between the basal and final values for each variable at each time point reach the minimum established according to the psychometric properties referring to the minimum clinical importance difference or minimum detectable change with a CI of 95%, if they are available in the literature. If they had been reported by different studies, it would be based on the suggested minimum value.

A post-hoc power analysis and an estimate of the effect size will be implemented with the tool G*Power [20], based on Cohen’s *d* and considering a low, medium or high effect as a function of the result [30].

#### Dealing with missing data

Every effort will be made to reduce sample loss, particularly with regard to mid-term monitoring (1y). In the event that a participant cannot or decides not to attend the follow-up evaluation, it is proposed to resolve the self-reported surveys at home and forward them via email, or the possibility of the evaluator’s displacement is also granted. To deal with missing data, we will compare the baseline characteristics of those patients who have and have not been evaluated in search of potential sources bias in the results of the complete analyses. It is also intended to obtain a record of the reasons for drop-out and then evaluate the best mechanism of data analysis, with methods to impute data and reanalyze it with the intention to treat, and thus evaluate the possible impact on the conclusions [31].

#### Economic evaluation

An economic analysis will be carried out to evaluate the cost-effectiveness of the experimental proposals versus the non-active comparator proposal. This will take into consideration all costs that are potentially related to each intervention, to the resources related to the training of the participant in the hospital, or in the case of performing the intervention at home, related to education and the time invested in monitoring the participant in order to ensure the compliance with the treatment. Appropriate unit costs will be allocated according to data from the national health system, including not only the time spent by physiotherapists but also by physicians [32].

The measure of effectiveness will be based on the results of the EuroQoL-5D, which will be used to estimate Quality Adjusted Life Years (QALY) estimated with the Spanish intervention rate associated with the time trade-off method. The analysis will be based on the mean overall cost and mean overall effect of each intervention. If a technique is less costly and more effective it will be deduced that it is cost-effective and therefore the one that will best optimize the use of resources. The acceptability curve will be plotted for the estimation of uncertainties. Finally a sensitivity analysis will allow to know the robustness.

#### Plan to share and analyze data

A clinical trial evaluating the effects of a preoperative balance training which is supervised in the hospital versus a conventional preoperative training is being conducted in parallel at the Hospital Clínico y Universitario of Valencia (NCT-02995668). The design of the experimental intervention coincides with that of this work. For this reason, and if the 2 trials are successfully completed, we plan to share the data in order to compare the matching interventions (PRE_EXP1 versus PRE_NA). The analysis shall take into account the center effect or correlation between the data, which will be evaluated as intraclass correlation coefficients. Since the idea is to extend the results to the centers that are concerned by the experimental treatment, the analysis of data will involve a mixed effects regression model, and treatment condition, covariates and other cofounding factors may be included as fixed effects [33]. The combination of data from the experimental groups will make it possible to consider the potential influences due to the center effect, and is expected to provide results of greater consistency, external validity and generalizability [34].

## Discussion

Total knee replacement is a surgical procedure with a high prevalence and associated health costs. The main objective is the elimination of pain and the maximization of the knee function. It is applied as the solution of severe osteoarthritis, a condition that greatly affects the proprioceptive system of the joint. The surgery involves greater deterioration, even when the posterior cruciate is preserved. Both the degeneration due to prolonged osteoarthritis and the procedure bears the consequence that, even after surgery, many limitations often persist [3, 4]. This bears a clinical impact on the balance abilities of the patient, which may be a direct cause of the functional limitations found [11, 12].

An early rehabilitation is essential, a continuous treatment when the subjects tolerate higher intensities is beneficial, and even when a muscular preconditioning is always recommended before any highly invasive surgery, there is still controversy about the effects in the total knee replacement outcomes. This is especially applicable if a cost-effective procedure is sought. For this reason, the use of a specific preoperative program have to be justified in economic terms, given the limitation of resources [7].

The design of this research will determine the effects and benefits of conducting training oriented towards balance and proprioception recovery, but also whether an in-home training can be as effective as a hospital supervised preparative training. While it is true that the inclusion of a control group renders the project implementation more arduous, it also makes it possible to determine the real effects of the experimental interventions. In addition, the costs of the in-home program are considerably lower than those of the hospital care. Therefore, a more cost-effective solution will be found if it is shown that in-home training achieves similar effects to the supervised one. The analysis will allow to ultimately conclude if, indeed, implementation costs are justified, and the proposal is applicable to clinical practice.

The design of the interventions has been realistic, and the information provided by clinicians with long experience and by the patient feedback has been fundamental. The experimental intervention at home (PRE_EXP2) has been designed to cover the basic aspects of the one performed in the hospital (PRE_EXP1). Certainly the possibilities of supervised work and hospital instrumentation are not available at home, but this has been taken into account and the adaptation of the intervention was made considering a pragmatic design.

Inclusion criteria are broad, not restricted to a large number of patients with comorbidities, which will increase the applicability of the proposal. The exclusion criteria have been proposed not to promote the results, but only for reasons of safety in the implementation of interventions. Therefore there is no exclusion due to social disadvantages, depression, anxiety or poor motivation, which are often frequent.

The trial began the recruitment of participants in March 2017. We expect to finalize the recruitment by June 2018. Early-postoperative outcome data are expected to be available from October 2018. Follow-up evaluations will be performed during 2019. The trial is expected to be completed by the end of 2019.

This work will serve as a basis for reducing implementation bias. In case of substantial changes in the protocol, they will be duly reported with the reasons.

It is necessary to recognize limitations such as the fact that the follow-up is prolonged beyond usual in clinical practice, and that some of the measures are not always used by clinicians, although they are indeed used in research. We plan to share, critically appraise and disseminate the results by offering critical discussion of the findings, description of potential clinical impact, clinical application, and contextualization within contemporary literature.

## Abbreviations

KOOS: Knee Injury Osteoarthritis Outcome Score
PRE_EXP1: In-hospital preoperative intervention
PRE_EXP2: In-home preoperative intervention
PRE_NA: Non-active preoperative intervention
QALY: Quality Adjusted Life Years
ROM: Range of Movement
